# Supplementary material for: RNA three-dimensional structure drives the sequence organization of potato spindle tuber viroid quasispecies
Source: PLoS Pathog. 2024 Apr 4;20(4):e1012142. doi: 10.1371/journal.ppat.1012142 (PMC11020406; doi:10.1371/journal.ppat.1012142)
Supplement: S1 Table — Samples were collected for deep sequencing at three sites, as illustrated in Fig 1C, namely IR (inoculated region), LM (margin of inoculated leaves), and Sys (systemic leaves). Three biological replicates (Reps) were included. Additionally, the pool sample used for the initial agroinoculation was also subjected to deep sequencing. After removing unrelated regions, at least 23,000 reads were obtained for each library. For each, the number of total reads, read number of wild type (WT), number of unique sequences, and sequence diversity (normalized Shannon entropy) are presented. IR and Sys samples are shown with yellow background. (DOCX) [file ppat.1012142.s001.docx]

**S1 Table Data summary of 70 deep sequencing libraries derived from three stem mutant pools and four loop mutant pools.**

| Regions | Samples | Reps | Total number of reads | Reads of WT | Number of unique sequences | Diversity (Normalized Shannon entropy) |
| --- | --- | --- | --- | --- | --- | --- |
| Stem 3 | Pool |  | 64980 | 1125 | 1177 | 0.972307336 |
|  | IR | R1 | 37518 | 12380 | 1283 | 0.718652107 |
|  |  | R2 | 46375 | 16021 | 1196 | 0.698759539 |
|  |  | R3 | 37006 | 8712 | 1316 | 0.729844482 |
|  | LM | R1 | 35301 | 28016 | 474 | 0.22458508 |
|  |  | R2 | 31435 | 24070 | 605 | 0.262032174 |
|  |  | R3 | 27692 | 22607 | 685 | 0.22603989 |
|  | Sys | R1 | 53654 | 44507 | 398 | 0.193813631 |
|  |  | R2 | 54226 | 42935 | 312 | 0.215997511 |
|  |  | R3 | 41113 | 35708 | 238 | 0.155727075 |
| Stem 15 | Pool |  | 61877 | 632 | 1148 | 0.976661202 |
|  | IR | R1 | 51104 | 14551 | 1354 | 0.692292556 |
|  |  | R2 | 47757 | 17546 | 1330 | 0.636355696 |
|  |  | R3 | 53729 | 16754 | 1396 | 0.668014734 |
|  | LM | R1 | 48366 | 37987 | 1004 | 0.225317721 |
|  |  | R2 | 36694 | 31739 | 845 | 0.151102712 |
|  |  | R3 | 63083 | 49355 | 972 | 0.211058618 |
|  | Sys | R1 | 50510 | 43155 | 208 | 0.182857253 |
|  |  | R2 | 45131 | 40508 | 195 | 0.134665583 |
|  |  | R3 | 50178 | 45910 | 207 | 0.105960459 |
| Stem 26 | Pool |  | 60797 | 2728 | 332 | 0.940126265 |
|  | IR | R1 | 42323 | 12561 | 472 | 0.676203754 |
|  |  | R2 | 38094 | 12305 | 490 | 0.669560506 |
|  |  | R3 | 23252 | 10395 | 428 | 0.582418196 |
|  | LM | R1 | 44927 | 35622 | 220 | 0.254213192 |
|  |  | R2 | 38270 | 31743 | 162 | 0.198856986 |
|  |  | R3 | 33610 | 28434 | 271 | 0.199016478 |
|  | Sys | R1 | 53513 | 52125 | 131 | 0.043171387 |
|  |  | R2 | 70160 | 59849 | 166 | 0.166227204 |
|  |  | R3 | 60500 | 53053 | 176 | 0.14833716 |
| Loop 1 | Pool |  | 76113 | 1533 | 298 | 0.970185796 |
|  | IR | R1 | 54194 | 10653 | 276 | 0.867065738 |
|  |  | R2 | 53343 | 11664 | 270 | 0.849278994 |
|  |  | R3 | 55980 | 12415 | 284 | 0.835101812 |
|  | LM | R1 | 50251 | 41584 | 97 | 0.195053626 |
|  |  | R2 | 47334 | 38934 | 118 | 0.19390964 |
|  |  | R3 | 32792 | 25532 | 112 | 0.261512852 |
|  | Sys | R1 | 49251 | 45017 | 50 | 0.125294883 |
|  |  | R2 | 55662 | 50470 | 51 | 0.133124998 |
|  |  | R3 | 60835 | 52128 | 143 | 0.169408598 |
| Loop 6 | Pool |  | 76485 | 1585 | 4434 | 0.975328422 |
|  | IR | R1 | 57552 | 11233 | 4436 | 0.800844327 |
|  |  | R2 | 55669 | 11050 | 4480 | 0.766108578 |
|  |  | R3 | 54224 | 11014 | 4458 | 0.736364586 |
|  | LM | R1 | 36221 | 22795 | 1784 | 0.320580773 |
|  |  | R2 | 39644 | 23410 | 770 | 0.353463437 |
|  |  | R3 | 43095 | 31611 | 2045 | 0.243717345 |
|  | Sys | R1 | 55649 | 52425 | 290 | 0.073736437 |
|  |  | R2 | 49511 | 43066 | 356 | 0.135641641 |
|  |  | R3 | 48762 | 42319 | 505 | 0.146348562 |
| Loop 15 | Pool |  | 62509 | 2121 | 4102 | 0.971001851 |
|  | IR | R1 | 61053 | 12324 | 4114 | 0.748220817 |
|  |  | R2 | 62254 | 12453 | 4106 | 0.748031032 |
|  |  | R3 | 52520 | 8367 | 4100 | 0.781077904 |
|  | LM | R1 | 61851 | 44783 | 2105 | 0.26391686 |
|  |  | R2 | 44917 | 33507 | 1765 | 0.25144329 |
|  |  | R3 | 49914 | 35442 | 2555 | 0.289284424 |
|  | Sys | R1 | 62120 | 53203 | 1464 | 0.142922459 |
|  |  | R2 | 66107 | 58389 | 579 | 0.123107525 |
|  |  | R3 | 43871 | 38592 | 647 | 0.133090035 |
| Loop 27 | Pool |  | 73628 | 2902 | 343 | 0.915344445 |
|  | IR | R1 | 56701 | 8762 | 343 | 0.803969923 |
|  |  | R2 | 61106 | 7554 | 344 | 0.82990161 |
|  |  | R3 | 52904 | 6898 | 347 | 0.814703163 |
|  | LM | R1 | 30014 | 24693 | 245 | 0.231117113 |
|  |  | R2 | 33076 | 29985 | 270 | 0.1373706 |
|  |  | R3 | 38709 | 35597 | 225 | 0.121050768 |
|  | Sys | R1 | 62183 | 50044 | 216 | 0.126249232 |
|  |  | R2 | 65429 | 51500 | 245 | 0.155766481 |
|  |  | R3 | 50836 | 47444 | 248 | 0.089754292 |
