# Supplementary material for: RNA three-dimensional structure drives the sequence organization of potato spindle tuber viroid quasispecies
Source: PLoS Pathog. 2024 Apr 4;20(4):e1012142. doi: 10.1371/journal.ppat.1012142 (PMC11020406; doi:10.1371/journal.ppat.1012142)
Supplement: S3 Table — Through the methods described in the legend of Fig 2D, mutated base pairs formed by two strands of the same stem in all three biological replicates (Reps) of each sample were analyzed. The weighted number of mutated group 1 or group 2 base pairs, and the ratio of the number of group 2 base pairs to the number of group 1 base pairs (weighted) were calculated and presented. IR and Sys samples are shown with yellow background. (DOCX) [file ppat.1012142.s003.docx]

**S3 Table Analysis of mutated base pair types in PSTVd quasispecies derived from stem mutant pools.**

| Regions | Samples | Reps | Weighted group 1 base pairs numbers | Weighted group 2 base pairs numbers | Group 2/group 1 ratio (weighted) |
| --- | --- | --- | --- | --- | --- |
| Stem 3 | Pool |  | 80886 | 158939 | 1.964975397 |
|  | IR | R1 | 38050 | 53334 | 1.401681997 |
|  |  | R2 | 43648 | 62647 | 1.435277676 |
|  |  | R3 | 43265 | 49262 | 1.138610886 |
|  | LM | R1 | 13377 | 6427 | 0.480451521 |
|  |  | R2 | 13781 | 9103 | 0.66054713 |
|  |  | R3 | 8167 | 7568 | 0.926656055 |
|  | Sys | R1 | 16153 | 8224 | 0.509131431 |
|  |  | R2 | 18775 | 7194 | 0.383169108 |
|  |  | R3 | 7778 | 4126 | 0.530470558 |
| Stem 15 | Pool |  | 77106 | 150972 | 1.957979924 |
|  | IR | R1 | 64702 | 60612 | 0.936787116 |
|  |  | R2 | 47671 | 53353 | 1.119191962 |
|  |  | R3 | 58999 | 65619 | 1.112205292 |
|  | LM | R1 | 15832 | 11219 | 0.708628095 |
|  |  | R2 | 7171 | 4846 | 0.675777437 |
|  |  | R3 | 23920 | 10724 | 0.448327759 |
|  | Sys | R1 | 13458 | 5137 | 0.381706048 |
|  |  | R2 | 7390 | 2553 | 0.345466847 |
|  |  | R3 | 5679 | 1590 | 0.27997887 |
| Stem 26 | Pool |  | 58529 | 117829 | 2.013172957 |
|  | IR | R1 | 35441 | 42145 | 1.189159448 |
|  |  | R2 | 32526 | 38510 | 1.183975896 |
|  |  | R3 | 15840 | 18209 | 1.149558081 |
|  | LM | R1 | 14748 | 8083 | 0.548074315 |
|  |  | R2 | 9427 | 3933 | 0.417205898 |
|  |  | R3 | 7616 | 4916 | 0.645483193 |
|  | Sys | R1 | 1305 | 173 | 0.13256705 |
|  |  | R2 | 14560 | 4863 | 0.333997253 |
|  |  | R3 | 11446 | 3768 | 0.329197973 |
