# Supplementary material for: RNA three-dimensional structure drives the sequence organization of potato spindle tuber viroid quasispecies
Source: PLoS Pathog. 2024 Apr 4;20(4):e1012142. doi: 10.1371/journal.ppat.1012142 (PMC11020406; doi:10.1371/journal.ppat.1012142)
Supplement: S4 Table — Using the methods described in the legend of Fig 4D, the mutated loop sequences from all three biological replicates (Reps) of each sample were aligned to their corresponding structural models using JAR3D. The JAR3D output cutoff values were then utilized to calculate the weighted average cutoff scores. IR and Sys samples are shown with yellow background. (DOCX) [file ppat.1012142.s004.docx]

**S4 Table Analysis of the weighted average cutoff scores of mutated loop sequences in the PSTVd quasispecies derived from mutant pools of selected loop regions.**

| Regions | Samples | Reps | Weighted average cutoff score |
| --- | --- | --- | --- |
| Loop 1 | Pool |  | 18.32931661 |
|  | IR | R1 | 21.75667392 |
|  |  | R2 | 23.09244575 |
|  |  | R3 | 23.41595991 |
|  | LM | R1 | 41.43811391 |
|  |  | R2 | 44.58488584 |
|  |  | R3 | 27.73979096 |
|  | Sys | R1 | 65.40310027 |
|  |  | R2 | 63.92335607 |
|  |  | R3 | 50.49646852 |
| Loop 6 | Pool |  | -87.38747314 |
|  | IR | R1 | -39.89037742 |
|  |  | R2 | -46.53112438 |
|  |  | R3 | -16.74762162 |
|  | LM | R1 | 4.469524874 |
|  |  | R2 | 13.26370683 |
|  |  | R3 | 9.343929664 |
|  | Sys | R1 | 45.69684865 |
|  |  | R2 | 51.58295113 |
|  |  | R3 | 37.06148406 |
| Loop15 | Pool |  | -22.39626567 |
|  | IR | R1 | 3.360487554 |
|  |  | R2 | 3.785078747 |
|  |  | R3 | 4.059253215 |
|  | LM | R1 | 29.16637685 |
|  |  | R2 | 24.49532146 |
|  |  | R3 | 22.54596301 |
|  | Sys | R1 | 33.33019118 |
|  |  | R2 | 40.71823468 |
|  |  | R3 | 37.57356027 |
| Loop 27 | Pool |  | -12.1846085 |
|  | IR | R1 | 2.636350795 |
|  |  | R2 | 0.929615027 |
|  |  | R3 | 2.849443308 |
|  | LM | R1 | 11.46111729 |
|  |  | R2 | 7.256517434 |
|  |  | R3 | 7.256517434 |
|  | Sys | R1 | 43.35619917 |
|  |  | R2 | 17.48380246 |
|  |  | R3 | 29.29590631 |
