# Supplementary material for: RNA three-dimensional structure drives the sequence organization of potato spindle tuber viroid quasispecies
Source: PLoS Pathog. 2024 Apr 4;20(4):e1012142. doi: 10.1371/journal.ppat.1012142 (PMC11020406; doi:10.1371/journal.ppat.1012142)
Supplement: S5 Table — In a previous study, the trafficking ability of multiple L6 mutants was analyzed [27]. Their occurrence in Sys samples of the present study was determined. Trafficking-competent mutants are indicated in red, while the trafficking-defective mutants detected in the pool sample and all three Sys sample replicates (Sys-rep1, Sys-rep2, and Sys-rep3) are shown in yellow background. (DOCX) [file ppat.1012142.s005.docx]

**S5 Table Analysis of the presence of previously functionally characterized loop 6 mutants in the Sys samples of the present study.**

| Sequences | Capable of trafficking | Cutoff score | Reads number | | | |
| --- | --- | --- | --- | --- | --- | --- |
|  |  |  | Pool | Sys-rep1 | Sys-rep2 | Sys-rep3 |
| UGAACGGGAG | No | 95.05799 | 12 | 3 | 5 | 5 |
| UGAUCGUGAG | No | -25.589739 | 21 | NA | NA | NA |
| UGACCGAGAG | No | 51.821884 | 21 | 50 | 37 | 187 |
| UGCCCGCUAG | No | 17.452663 | 7 | 1 | 2 | 4 |
| UGGCCGCUAG | No | 3.773364 | 14 | NA | NA | NA |
| UAACCGCGUG | No | -1.550459 | 13 | NA | NA | NA |
| UGACCGCGCG | No | 2.179023 | 11 | 13 | 7 | 14 |
| UUACCGCGCG | No | 18.16115 | 15 | 1 | 1 | 5 |
| UCACCGCGAG | Yes | 47.65543 | 8 | 354 | 713 | 605 |
| UGCCCGCGAG | No | 3.773364 | 11 | 18 | 11 | 28 |
| UCACCGCGCG | No | 16.953888 | 9 | NA | 4 | 3 |
| UUACCGCGGG | No | -29.399923 | 10 | NA | 5 | NA |
| UCACCGCGGG | No | -29.399923 | 12 | 1 | 2 | NA |
| UAACCGCGAG | Yes | 50.16498 | 7 | 350 | 751 | 609 |
| UUACCGCGAG | Yes | 53.99573 | 17 | 377 | 687 | 672 |
| UAACCGCGCG | No | 19.007675 | 13 | 1 | 1 | 1 |
| UGACCGCUAG | No | 49.38787 | 6 | 39 | 16 | 40 |
| UCACCGCGUG | No | -29.399923 | 12 | NA | 1 | NA |
| UGAGCGUGAG | No | 31.262243 | 20 | NA | 3 | 1 |
| UGACCGUGAG | No | 51.7561 | 26 | 30 | 35 | 41 |
| UGAGCGGGAG | No | 70.88035 | 14 | 11 | 30 | 21 |
| UGAUCGAGAG | No | 3.130295 | 22 | NA | NA | NA |
| UGUCCGCUAG | No | -27.805584 | 13 | NA | NA | NA |
| UGAGCGCGAG | No | 5.989208 | 25 | 7 | 18 | 9 |
| UGAGCGAGAG | No | 5.989208 | 11 | NA | NA | NA |
| UGAUCGGGAG | No | 52.78912 | 27 | 9 | 29 | 209 |
| UGACCGCGUG | No | 46.312637 | 8 | 71 | 40 | 75 |
| UGAUCGCGAG | No | 5.989208 | 26 | 21 | 8 | 16 |
| UGAACGUGAG | No | 52.40605 | 17 | 8 | 31 | 29 |
| UGCCCGCAAG | No | 43.334904 | 6 | 1 | 3 | 1 |
| UGUCCGCGAG | No | 43.98662 | 21 | 15 | 6 | 21 |
| UGGCCGCGAG | No | 72.84788 | 13 | 33 | 45 | 39 |
| UGUCCGCCAG | No | -4.574855 | 21 | NA | NA | NA |
| UGGCCGCCAG | No | 3.773364 | 16 | NA | NA | NA |
| UAACCGCGGG | No | -29.399923 | 21 | NA | 3 | NA |
| UGCCCGCCAG | No | 14.378858 | 8 | NA | NA | NA |
| UGACCGGGAG | No | 5.989208 | 11 | 23 | 26 | 39 |
| UGUCCGCAAG | No | 35.632565 | 12 | 1 | 1 | NA |
| UGAACGAGAG | No | 51.30386 | 14 | 1 | 4 | 7 |
| UGGCCGCAAG | No | 3.773364 | 7 | NA | NA | NA |
| UGACCGCCAG | No | 50.058258 | 7 | 3 | 5 | 7 |
| UUACCGCGUG | No | -29.399923 | 5 | NA | 2 | 2 |
| UGAACGCGAG | No | 52.681946 | 9 | 29 | 43 | 41 |
| UGACCGCAAG | Yes | 87.175285 | 1 | 809 | 2129 | 1184 |
| UGACCGCGGG | No | 19.767725 | 19 | 9 | 13 | 16 |
