# Supplementary material for: RNA three-dimensional structure drives the sequence organization of potato spindle tuber viroid quasispecies
Source: PLoS Pathog. 2024 Apr 4;20(4):e1012142. doi: 10.1371/journal.ppat.1012142 (PMC11020406; doi:10.1371/journal.ppat.1012142)
Supplement: S6 Table — The trafficking ability of selected loop 15 mutants was previously analyzed [31]. Their occurrence in Sys samples of the present study was determined. Trafficking-competent mutants are indicated in red, while the trafficking-defective mutants detected in the pool sample and all three Sys sample replicates (Sys-rep1, Sys-rep2, and Sys-rep3) are shown in yellow background. (DOCX) [file ppat.1012142.s006.docx]

**S6 Table The presence of previously functionally characterized loop 15 mutants in the Sys samples of the present study.**

| Sequences | Capable of trafficking | Cutoff score | Reads number | | | |
| --- | --- | --- | --- | --- | --- | --- |
|  |  |  | Pool | Sys-rep1 | Sys-rep2 | Sys-rep3 |
| GGAAACCGCCACUAC | Yes | 45.377388 | 46 | 23 | 29 | 1 |
| GGAAACCGCUUCUAC | No | 6.920692 | 48 | 33 | 17 | 4 |
| GGAAACCGCUAUGAC | No | -14.85611 | 23 | NA | NA | NA |
| GGAAACCGCUAUCAC | No | 18.680075 | 12 | 1 | 4 | 2 |
| GGAAACCGCUAUAAC | No | 14.71032 | 13 | 2 | 1 | 3 |
| GGAAACCGCUAGCAC | No | 21.414446 | 14 | 2 | 5 | 3 |
| GGAAACCGCUAGAAC | No | 38.687805 | 11 | 4 | 4 | 4 |
| GGAAACCGCUACGAC | No | -14.85611 | 327 | 269 | 260 | NA |
| GGAAACCGCGACUAC | Yes | 47.59106 | 93 | 64 | 69 | 6 |
| GGAAACCGCUACUUC | No | 29.20407 | 55 | 38 | 40 | 4 |
| GGAAACCGCUACAAC | No | 13.171974 | 24 | 11 | 14 | 3 |
| GGAAACCGCUAGGAC | No | 32.14022 | 12 | 5 | 5 | 5 |
| GGAAACCGCUACCAC | No | 19.222498 | 30 | 29 | 22 | 2 |
| GGAAACCGCAACUAC | Yes | 29.991385 | 14 | 10 | 5 | 5 |
| GGAAACCGCUAGUAC | Yes | 74.58829 | 17 | 535 | 430 | 265 |
| GGAAACCGCUGCUAC | No | 5.374806 | 22 | 6 | 5 | NA |
| GGAAACCGCUCCUAC | No | 19.727196 | 91 | 45 | 48 | 2 |
| GGAAACCGCUAUUAC | Yes | 47.45463 | 1019 | 2224 | 2582 | 1672 |
| GGAAACCGCUAAAAC | No | 14.633335 | 17 | 4 | 5 | 6 |
| GGAAACCGCUAACAC | No | 18.727081 | 9 | 5 | 4 | 2 |
| GGAAACCGCUAAGAC | No | -7.31757 | 9 | NA | 1 | NA |
| GGAAACCGCUACUGC | No | 14.932714 | 27 | 13 | 25 | 3 |
| GGAAACCGCUAAUAC | Yes | 46.601353 | 21 | 537 | 421 | 256 |
| GGAAACCGCUACUCC | No | 6.998099 | 142 | 114 | 98 | NA |
