# Supplementary material for: RNA three-dimensional structure drives the sequence organization of potato spindle tuber viroid quasispecies
Source: PLoS Pathog. 2024 Apr 4;20(4):e1012142. doi: 10.1371/journal.ppat.1012142 (PMC11020406; doi:10.1371/journal.ppat.1012142)
Supplement: S7 Table — Our previous study analyzed the ability of selected loop 27 mutants in trafficking [29]. Their occurrence in Sys samples of the present study was determined. Trafficking-competent mutants are indicated in red, while the trafficking-defective mutants detected in the pool sample and all three Sys sample replicates (Sys-rep1, Sys-rep2, and Sys-rep3) are shown in yellow background. (DOCX) [file ppat.1012142.s007.docx]

**S7 Table The presence of previously functionally characterized loop 27 mutants in the Sys samples of the present study.**

| Sequences | Capable of trafficking | Cutoff score | Reads number | | | |
| --- | --- | --- | --- | --- | --- | --- |
|  |  |  | Pool | Sys-rep1 | Sys-rep2 | Sys-rep3 |
| UUGUCA | Yes | 45.292374 | 354 | 10400 | 234 | 467 |
| UUCUUA | No | 13.713426 | 465 | 5 | 9 | 10 |
| UUCUCA | Yes | 45.292374 | 171 | 14 | 14 | 16 |
| UUUUGA | Yes | 51.4796 | 421 | 14 | 33 | 13 |
| UUAUAA | No | -3.228026 | 572 | 6 | 14 | 13 |
| UGUUCA | No | 68.42105 | 241 | 22 | 65 | 81 |
| UUUAAA | No | 13.713426 | 409 | 3 | 10 | 17 |
| UGGUCA | No | 13.713426 | 274 | 4 | 17 | 5 |
| UUUCCA | No | 45.292374 | 141 | 14 | 16 | 15 |
| UCUUCA | No | 68.42105 | 70 | 6 | 8 | 10 |
