# Supplementary material for: RNA three-dimensional structure drives the sequence organization of potato spindle tuber viroid quasispecies
Source: PLoS Pathog. 2024 Apr 4;20(4):e1012142. doi: 10.1371/journal.ppat.1012142 (PMC11020406; doi:10.1371/journal.ppat.1012142)
Supplement: S8 Table — (DOCX) [file ppat.1012142.s008.docx]

**S8 Table** **Sequences of primers used in mutant pool preparation and library preparation.**

| Names | Sequence (5'-3') |  |
| --- | --- | --- |
| Stem3-F | CGGGGCGAGGGTGTTTAGCCCTTGNANNCNNAGTTGGTTCCTCGGAACTAAAC | Mutant pool preparation |
| Stem3-R | AAGGGCTAAACACCCTCGCCCCG |  |
| Stem15-F | CACCCCTCGCCCCCTTTGCGCTGTNGNNTNNGCTACTACCCGGTGGAAACAACTG |  |
| Stem15-R | GCGCAAAGGGGGCGAGGGGTG |  |
| Stem26-F | GTAATTCCCGCCGAAACAGGGTTTTCNNCNNTCCTTTCTTCGGGTGTCCTTCC |  |
| Stem26-R | CCCTGTTTCGGCGGGAATTAC |  |
| Loop1-F | CCCTTGGAACCGCAGTTGGTTCNNNNGAACTAAACTCGTGGTTCCTG |  |
| Loop1-R | CCAACTGCGGTTCCAAGG |  |
| Loop6-upper-F | CGTGGTTCCTGTGGTTCACACCTNNNCTCCTGAGCAGAAAAGAAAAAAG |  |
| Loop6-upper-R | TGTGAACCACAGGAACCAC |  |
| Loop6-lower-F | TTCTCTATCTTACTTGCTTCGGGGNNNGGGTGTTTAGCCCTTGGAAC |  |
| Loop6-lower-R | GAAGCAAGTAAGATAGAGAAAAAGCG |  |
| Loop15-F | CCCCTTTGCGCTGTCGCTTCGGNNNNNNCCCGGTGGAAACAACTGAAG |  |
| Loop15-R | AAGCGACAGCGCAAAGGG |  |
| Loop27-F | CCGCCGAAACAGGGTNNNNACCCTTCCTTTCTTCGGG |  |
| Loop27-R | CCCGAAGAAAGGAAGGGTNNNNACCCTGTTTCGGCGG |  |
| PSTVd-universal-F | GTGACCAGCTCGAATTTCCCC |  |
| PSTVd-universal-R | GGGGAAATTCGAGCTGGTCAC |  |
| PSVTd-F | GGGATCCCCGGGGAAACC | Library preparation |
| PSVTd-R | TGAAGCGCTCCTCCGAGCC |  |
