# Supplementary figures and images for: RNA three-dimensional structure drives the sequence organization of potato spindle tuber viroid quasispecies
Source: PLoS Pathog. 2024 Apr 4;20(4):e1012142. doi: 10.1371/journal.ppat.1012142 (PMC11020406; doi:10.1371/journal.ppat.1012142)

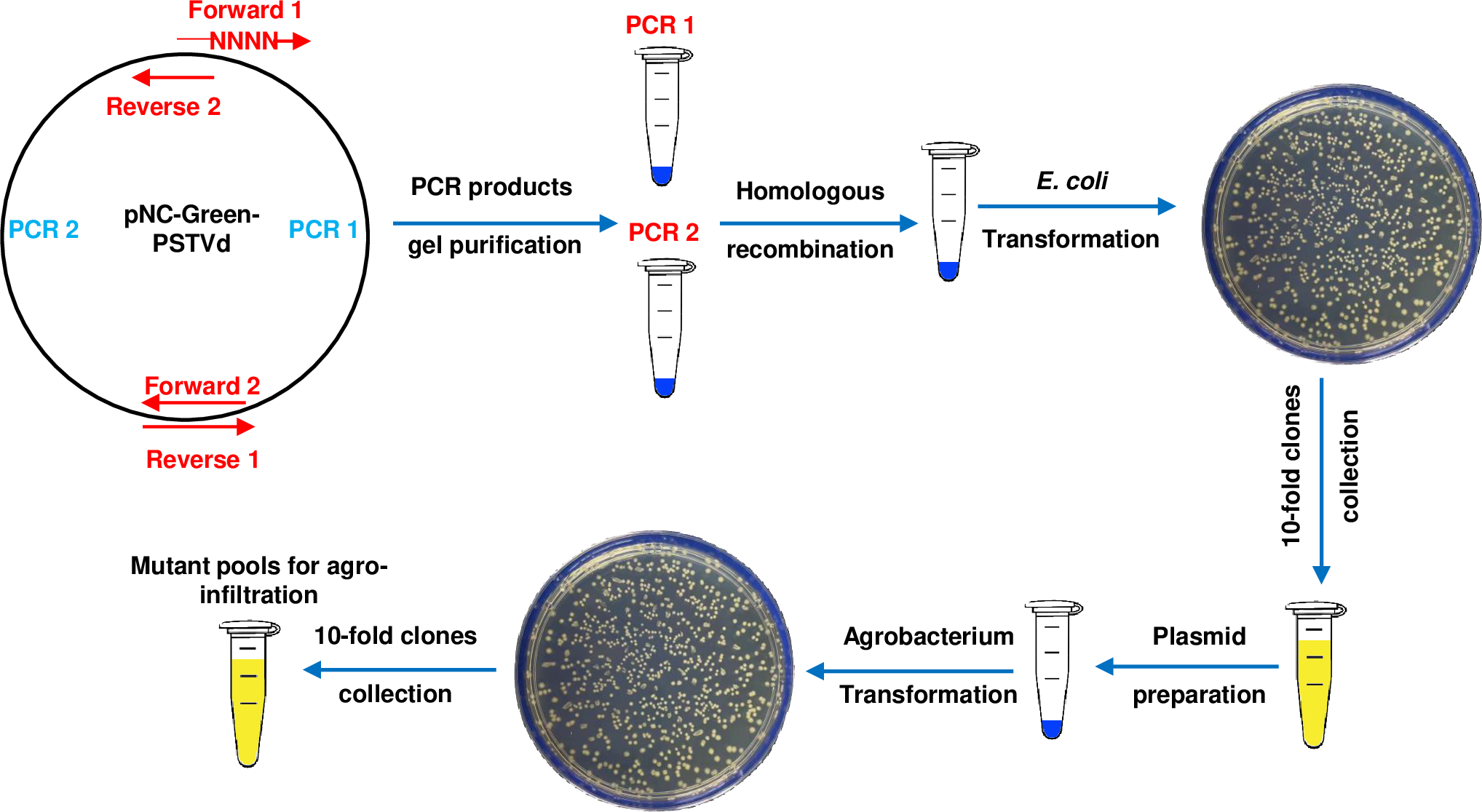

Supplement: S1 Fig — A pNC-Green vector, which expresses the plus-strand PSTVd intermediate strain (pNC-Green-PSTVd), was used as a template to create PSTVd mutant pools through PCR. To generate the mutant pools, two PCR reactions were performed. In PCR 1, a degenerate forward primer (Forward 1) covering the target region was used along with a regular reverse primer (Reverse 1). PCR 2 involved a pair of regular primers (Forward 2 and Reverse 2). Forward 2 and Reverse 1 were universal primers used in all PCRs. The resulting PCR products underwent homologous recombination, and the resulting products were transformed into competent E. coli cells. To cover all possible mutants, more than 10-fold clones were collected and combined, followed by plasmid preparation. The collected plasmids were then transformed into competent cells for Agrobacterium transformation. Finally, more than 10-fold clones were collected and mixed to prepare the mutant pools for agroinoculation. (TIF) [file ppat.1012142.s009.tif]

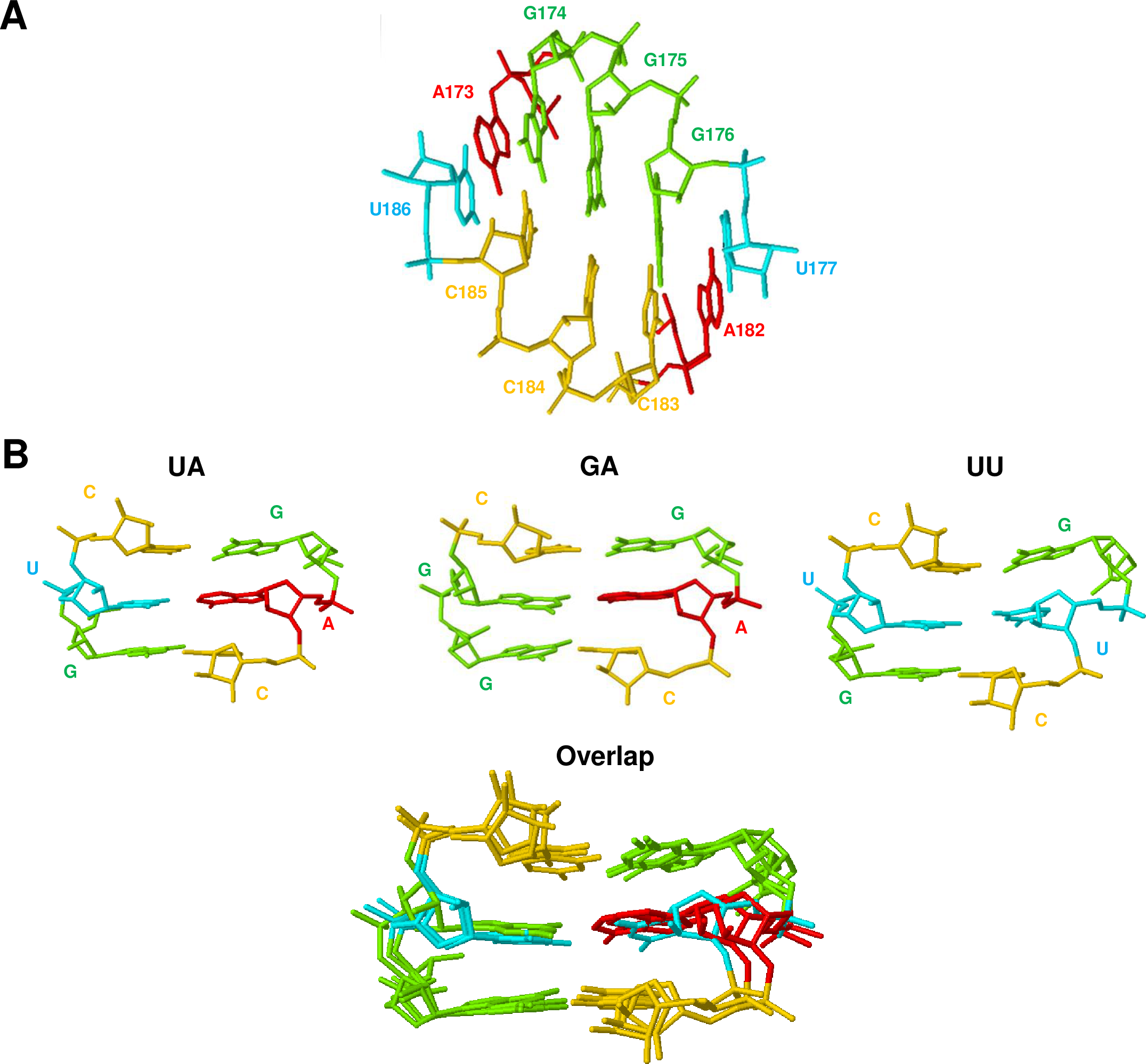

Supplement: S2 Fig — (A) The structural model of PSTVd stem 27 was obtained by querying the BGSU RNA site (http://rna.bgsu.edu/rna3dhub/). The model is sourced from PDB ID 1DQH. Positions 4 to 8 in chain A and positions 11 to 15 in chain B correspond to PSTVd positions 173 to 177 and 182 to 186, respectively. (B) The structural models of UA, GA, and UU enclosed by two GC cWW base pairs. These models were sourced from PDB ID 1DQH (positions 581 to 583 in chain A and positions 1261 to 1263 in chain B), PDB ID 4M4O (positions 12 to 14 in chain A and positions 46 to 48 in chain B), and PDB ID 4P43 (positions 28 to 30 in chain A and positions 19 to 21 in chain B), respectively. These models were overlapped to display the structural changes induced by replacing the UA cWW pair with GA or UU base combinations. (TIF) [file ppat.1012142.s010.tif]

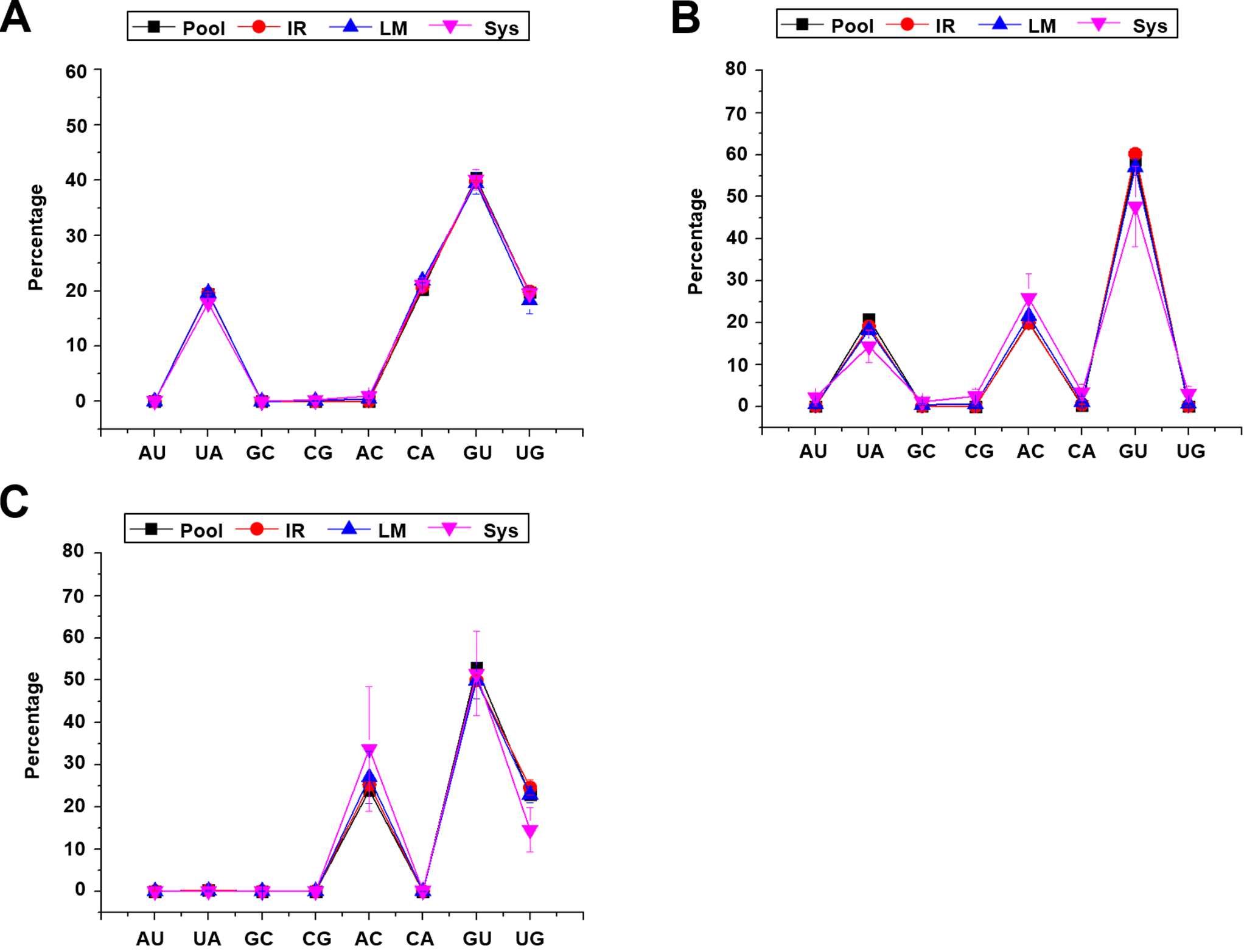

Supplement: S3 Fig — The reads for eight mutated purine/pyrimidine pairs (AU, UA, GC, CG, AC, CA, GU, and UG) in S3 (A), S15 (B), and S26 (C) libraries were calculated using the formula RC = Σ(N_i * R_i). Here, RC denotes the read count, N_i signifies the occurrences of each purine/pyrimidine pair in unique sequence i, and R_i represents the total reads for unique sequence i. The percentage of RC for each of the eight base pairs in the total RC of all purine/pyrimidine pairs was then computed. Data for IR, LM, and Sys are presented as mean ± SD of three biological replicates, while data for pool libraries are expressed as a single value. (TIF) [file ppat.1012142.s011.tif]

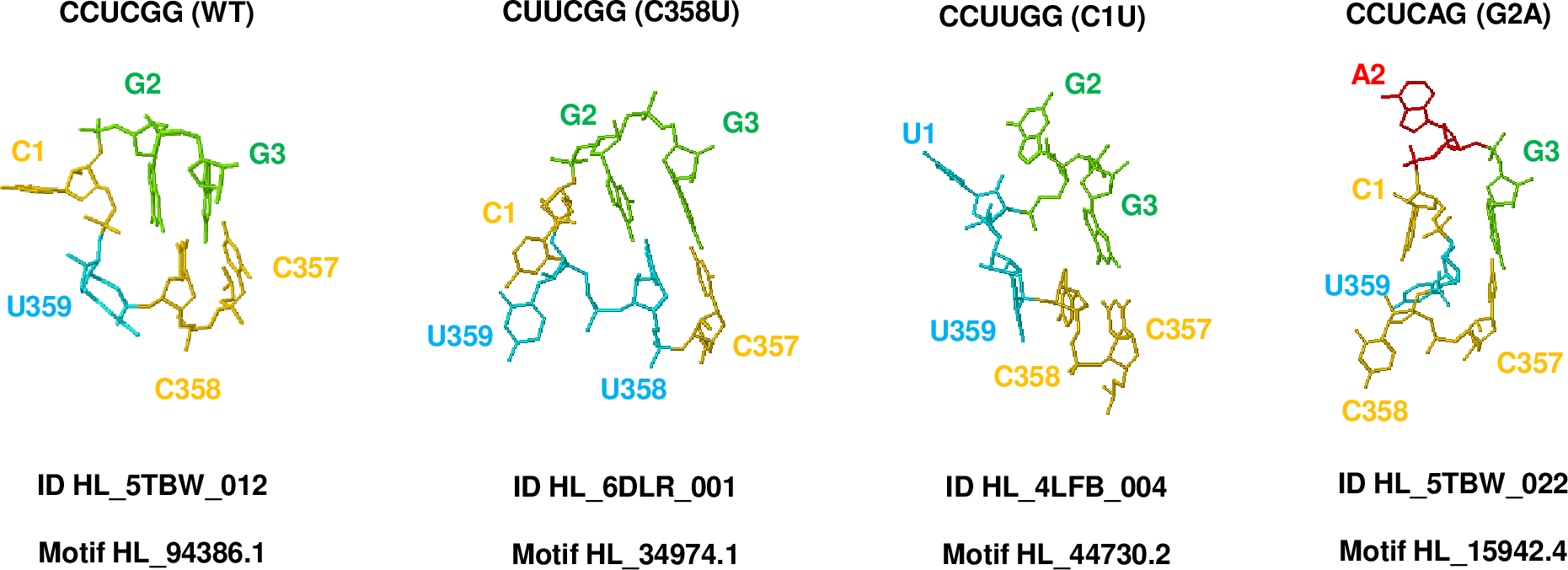

Supplement: S4 Fig — Structural models for both the WT L1 and its mutants, C358U, C1U, and G2A, were generated using JAR3D. The sequences for the WT and mutants were provided above the models. Below the models, the corresponding loop IDs from the BGSU RNA site (http://rna.bgsu.edu/rna3dhub/) and the names of the RNAs from which the models were derived were presented. (TIF) [file ppat.1012142.s012.tif]

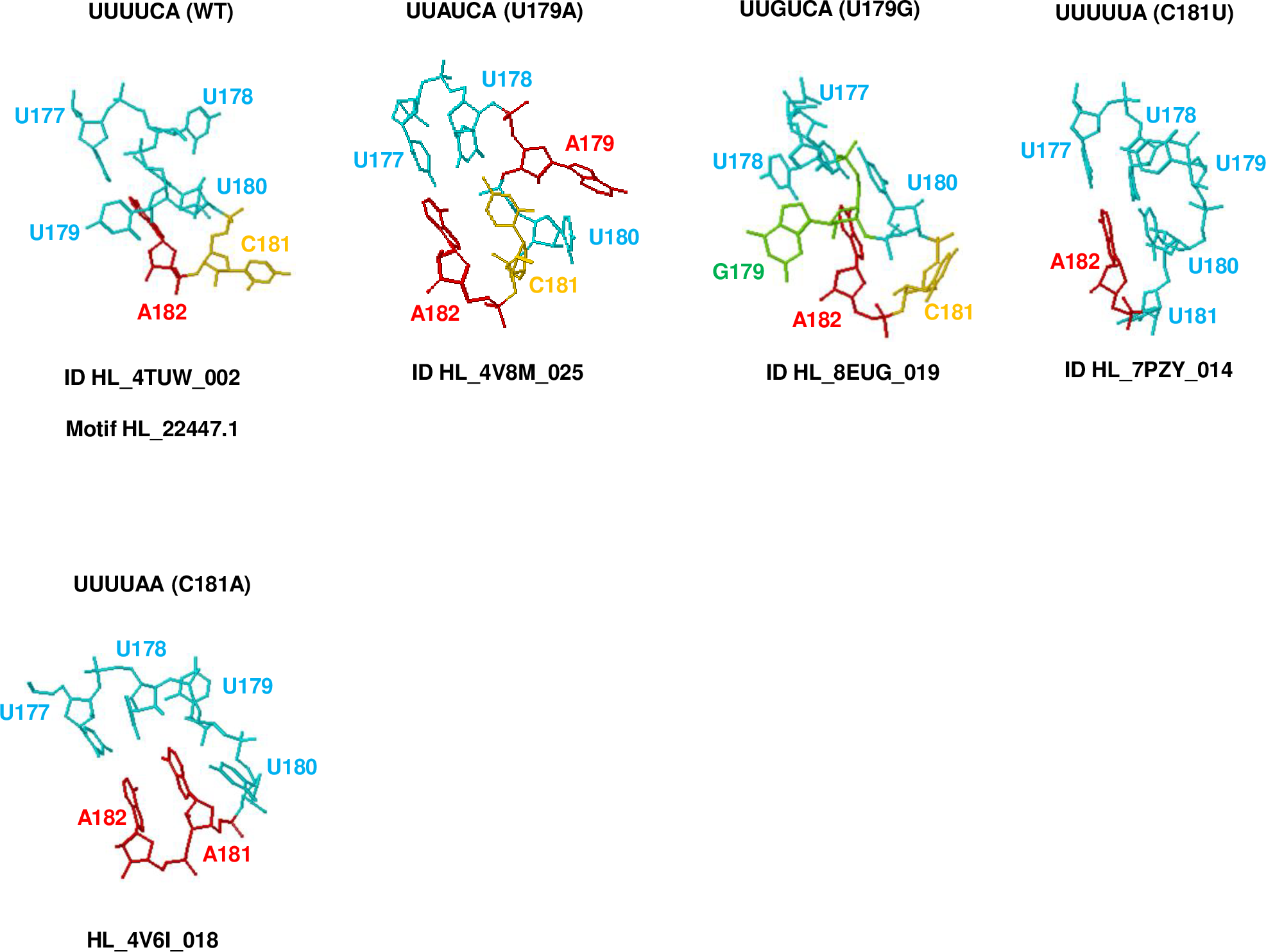

Supplement: S5 Fig — A structural model for the WT L27 was constructed using JAR3D. Additionally, structural models for four mutants (U179A, U179G, C181U, and C181A) were generated by directly querying the BGSU RNA group database (http://rna.bgsu.edu/rna3dhub/). The sequences for both the WT and mutants were provided above their respective models. Below the models, the corresponding loop IDs from the BGSU RNA site and the names of the RNAs from which the models were derived were listed for the WT. For the mutants, only the loop IDs were presented. (TIF) [file ppat.1012142.s013.tif]

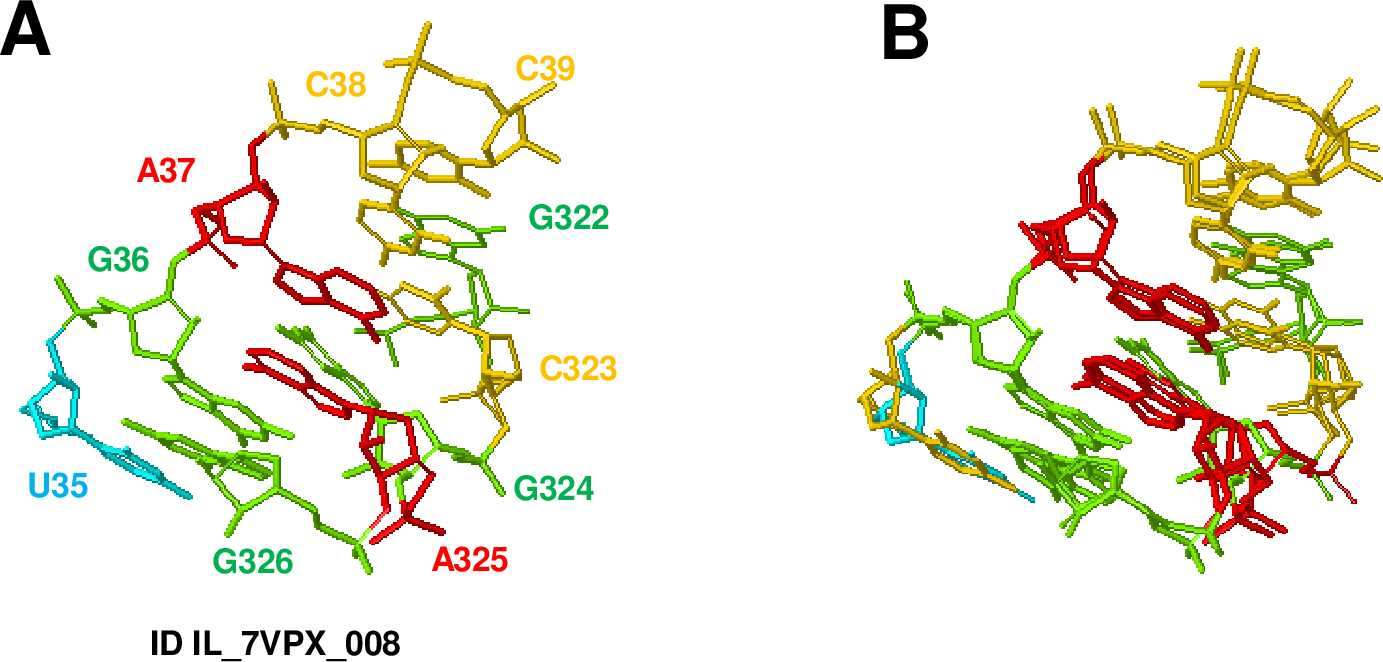

Supplement: S6 Fig — By directly querying the BGSU RNA group database (http://rna.bgsu.edu/rna3dhub/), a structural model (ID IL_7VPX_008) with the exact same sequence as L6 was obtained (A). This exact match superposes extremely well with the model of L6 presented in Fig 3 (B). (TIF) [file ppat.1012142.s014.tif]
