# Supplementary material for: RNA three-dimensional structure drives the sequence organization of potato spindle tuber viroid quasispecies
Source: PLoS Pathog. 2024 Apr 4;20(4):e1012142. doi: 10.1371/journal.ppat.1012142 (PMC11020406; doi:10.1371/journal.ppat.1012142)
Supplement: Attachment — Submitted filename: Responses to reviewers CLZ.docx [file ppat.1012142.s016.docx]

Dear editor and reviewers,

We appreciate the reviewers for their positive comments on our manuscript. In response to their feedback, we have diligently revised the manuscript. Specifically, we made the following modifications:

1) JAVA code used for data analysis has been uploaded to GitHub (<https://github.com/tomwu1495/PSTVdQuasispecies>)
2) Progeny sequence has been performed on WT group, and the data have been presented in Fig.5C.
3) The typo has been fixed.
4) We have presented the structural model of one stem (S26). Although no models with exactly the same sequence have been found for S3 or S15, (also no single mutants were found), an alternative method was used to illustrate how a single mutation affects the structure of a stem. Please refer to S2 Fig.
5) We presented structural models of several single mutants of L1 and L27.
6) During the revision of the manuscript, a model with exactly the same sequence was identified for L6. The exact match superposes extremely well with the model we present in Fig. 3. This model and the superposition are shown in S6 Fig.

Now, this manuscript contains six Supplementary Figs. The old S2 Fig. is now S3. A typo in Fig.1 (pairs, not paris) has been corrected.

Please check our responses for each comment below.

Reviewer's Responses to Questions

**Part I - Summary**
Please use this section to discuss strengths/weaknesses of study, novelty/significance, general execution and scholarship.

Reviewer #1: The revised version has been appropriately improved according to the comments by reviewers.
In particular, the authors performed additional co-infection experiments using trafficking-deficient mutants and WT to substantiate their proposal that some of the trafficking-defective mutants can be carried to the upper leaves by WT. This finding is new in viroid, which made the manuscript more attractive.

Reviewer #2: The authors have carefully and diligently addressed all of my queries.

Reviewer #3: Not applicable

**Part II – Major Issues: Key Experiments Required for Acceptance**
Please use this section to detail the key new experiments or modifications of existing experiments that should be absolutely required to validate study conclusions.

Generally, there should be no more than 3 such required experiments or major modifications for a "Major Revision" recommendation. If more than 3 experiments are necessary to validate the study conclusions, then you are encouraged to recommend "Reject".

Reviewer #1: None

Reviewer #2: (No Response)

Reviewer #3: In this revised version of their original manuscript Wu et al have addressed some of the issues of their first submission. Nevertheless, there are still some aspects that need to be addressed:

-The Java program used to analyze their sequencing data needs to be shared through Github (www.github.com) or any other repository for code.

Reply-

Line 613-614: The code is available at GitHub (<https://github.com/tomwu1495/PSTVdQuasispecies>).

-It is mentioned multiple times in the manuscript that some of the mutations studied affect PSTVd's 3D structure, yet, it is never shown how any of the mutations disrupt this. In the case of mutations in stems, the 3D structure of these stems is never shown. Could the authors please add to the manuscript: 1) the predicted 3D structure of the stems where mutations have been analyzed, and 2) the effect of some of the mutations on the predicted 3D structure for both stems and loops. At least some of the most dramatic alterations should be shown either in the main or supplementary figures.

Reply-

This is a very good suggestion.

Regarding the stem regions, the WebFR3D tool on the BGSU RNA site (http://rna.bgsu.edu/rna3dhub/) was searched to identify helices with identical sequences and base pairs as S3, S15, and S26. An exact match for S26 was found, and this model was presented in S2A Fig. However, no models with the same sequences were found for S15 and S26, or for the single mutants of these stems. These are long stems, and not all sequences of such stems have been observed in experimental 3D structures.

However, analysis of resolved structures of helices has shown that a single base substitution typically does not bring about significant alterations to the backbone structure. As an alternative approach, structural changes induced by replacing the UA cWW base pair, enclosed by two GC cWW base pairs, with GA or UU base combinations were presented to illustrate how a mutation can impact the overall structure of a stem. Please check S2B Fig. It was observed that all changes significantly affect the replaced site but have little impact on the surrounding base pairs. Nevertheless, minor alterations to the backbone can hold considerable biological relevance, as they can modify the helix's configuration and, consequently, its potential interactions with binding partners.

The following text has been added to the manuscript-

Lines 194-208: “Searches with the WebFR3D tool on the BGSU RNA site (http://rna.bgsu.edu/rna3dhub/) were conducted to identify helices with identical sequences and basepairs as S3, S15, and S26. An exact match for S26 was found (S2A Fig.). However, no matches or single mutation matches were found for S3 and S15. This outcome is not surprising, given that these stems are longer than the typical RNA double helix. Constructing a model for these stems would simply reflect the expectation that they will closely resemble other RNA helices of the same size. In fact, analysis of resolved structures of helices revealed that a single base substitution typically brings about no significant alterations to the backbone structure. As an alternative approach, structural changes induced by replacing the UA cWW base pair, enclosed by two GC cWW base pairs, with GA or UU base combinations were presented. As anticipated, these two substitutions significantly affect the replaced site while having minimal impact on the surrounding base pairs (S2B Fig.). Nonetheless, even minor alterations to the backbone can carry significant biological relevance, as they have the potential to modify the helix's configuration and, consequently, its interactions with binding partners.”

Regarding the loop regions, JAR3D was employed to search for structural models of single mutants within L1, L6, L15, and L27. Structural models for single mutants were obtained only for three single mutants of L1: C358U, C1U, and G2A, whereas no such models were found for single mutants of other loops using JAR3D. Please check S4 Fig. for structural models of L1 mutants.

Since JAR3D only targets x-ray RNA structures with a resolution higher than 3.5 Å, the BGSU RNA group database (<http://rna.bgsu.edu/rna3dhub/>) of loops was also queried to investigate structural models with identical sequences for single mutants of other loops, thereby extending the search to structures solved by NMR, by cryo-EM, or with resolutions worse than 3.5 Å. Models with the same sequences were discovered for four mutants of L27: U179A, U179G, C181U, and C181A (S5 Fig.). No such models were found for single mutants of L6 and L15. However, an exact match was found for L6 in a cryo-EM structure at 3.0A resolution. That will be noted below.

Drawing from the observations made in L1 and L27, we concluded that even single mutations can significantly influence the overall structure of loops.

The following text has been added to the manuscript-

Lines 404-418: “JAR3D was utilized to search for structural models of single mutants within L1, L6, L15, and L27. Structural models with identical sequences were obtained for three single mutants of L1: C358U, C1U, and G2A (S4 Fig.). However, no structural models with the same sequence were found for mutants of other loops. These three single mutations were noted to significantly impact the overall structure of L1. Since JAR3D only targets X-ray RNA structures with a resolution higher than 3.5 Å, the BGSU RNA group database (<http://rna.bgsu.edu/rna3dhub/>) of loops was also queried to investigate structural models with identical sequences for single mutants of other loops, thereby extending the search to structures solved by NMR, by cryo-EM, or with resolutions worse than 3.5 Å. Models with the same sequences were discovered for four mutants of L27: U179A, U179G, C181U, and C181A (S5 Fig.). Similar to observations from L1, these four mutations significantly impacted the structure of L27. In the case of L6, an exact match was discovered during revisions to the paper, which exactly matches the WT sequence of L6 (S6A Fig.). The exact match superposes extremely well with the model presented in Fig. 3 (S6B Fig.). No exact matches were found to L15, and no single mutation matches were found to L6 or L15.”

**Part III – Minor Issues: Editorial and Data Presentation Modifications**
Please use this section for editorial suggestions as well as relatively minor modifications of existing data that would enhance clarity.

Reviewer #1: The following point can be further improved.

L475-L481; As mentioned above (Part I; Summary), the data shown in the new Fig. 5 supported strongly the notion that WT PSTVd can help systemic spreading of some of the trafficking-defective mutants with similar 3D-structure. However, if the authors add the data from the control plot, namely plants infected with WT alone, the results will be even stronger.

Reply-

Following your suggestion, we sequenced PSTVd progeny in three plants of the WT group. Please check revised Fig.5.

Line 507-509, the following description has been added: “In the WT group, 24, 18, and 23 clones were sequenced for plants 1, 2, and 3, respectively. While novel mutations (in bold) were observed in all three plants, none of the four selected trafficking-defective mutants were detected.”

Minor point.
L445; trafficking-competent mutants, --- change comma to period.

Reply-

Line 475-476, the comma actually needs to be removed to read “although they generally accumulated to lower levels than trafficking-competent mutants (S5-S7 Tables).”

Reviewer #2: (No Response)

Reviewer #3: (No Response)

PLOS authors have the option to publish the peer review history of their article. If published, this will include your full peer review and any attached files.


If you choose “no”, your identity will remain anonymous but your review may still be made public.


**Do you want your identity to be public for this peer review?** For information about this choice, including consent withdrawal, please see our.

Reviewer #1: No

Reviewer #2: No

Reviewer #3: No

Figure Files:

While revising your submission, please upload your figure files to the Preflight Analysis and Conversion Engine (PACE) digital diagnostic tool, https://pacev2.apexcovantage.com. PACE helps ensure that figures meet PLOS requirements. To use PACE, you must first register as a user. Then, login and navigate to the UPLOAD tab, where you will find detailed instructions on how to use the tool. If you encounter any issues or have any questions when using PACE, please email us at [figures@plos.org](mailto:figures@plos.org).

Reply-

All figures are generated using PACE.

Data Requirements:

Please note that, as a condition of publication, PLOS' data policy requires that you make available all data used to draw the conclusions outlined in your manuscript. Data must be deposited in an appropriate repository, included within the body of the manuscript, or uploaded as supporting information. This includes all numerical values that were used to generate graphs, histograms etc.. For an example see here: <http://www.plosbiology.org/article/info%3Adoi%2F10.1371%2Fjournal.pbio.1001908#s5>.

Reply-

All data were either included within the body of the manuscript or uploaded as supporting information.

The deep sequencing data generated in this study has been deposited in the NCBI Sequence Read Archive (SRA) under the accession number PRJNA1049893.

Reproducibility:

To enhance the reproducibility of your results, we recommend that you deposit your laboratory protocols in protocols.io, where a protocol can be assigned its own identifier (DOI) such that it can be cited independently in the future. Additionally, PLOS ONE offers an option to publish peer-reviewed clinical study protocols. Read more information on sharing protocols at <https://plos.org/protocols?utm_medium=editorial-email&utm_source=authorletters&utm_campaign=protocols>

 Reply-

All protocols have been described clearly in the main body of the manuscript.

References:

Please review your reference list to ensure that it is complete and correct. If you have cited papers that have been retracted, please include the rationale for doing so in the manuscript text, or remove these references and replace them with relevant current references. Any changes to the reference list should be mentioned in the rebuttal letter that accompanies your revised manuscript. If you need to cite a retracted article, indicate the article’s retracted status in the References list and also include a citation and full reference for the retraction notice.

Reply-

All references have been checked.
