# Supplementary material for: RNA three-dimensional structure drives the sequence organization of potato spindle tuber viroid quasispecies
Source: PLoS Pathog. 2024 Apr 4;20(4):e1012142. doi: 10.1371/journal.ppat.1012142 (PMC11020406; doi:10.1371/journal.ppat.1012142)
Supplement: Attachment — Submitted filename: Responses to reviewer comments.docx [file ppat.1012142.s015.docx]

Dear editors and reviewers,

We express our gratitude for the positive attitude towards our manuscript and the invaluable comments provided by all reviewers. Upon considering the reviewers' suggestions, we have thoroughly revised the entire manuscript.

Specifically, we made the following major changes to the manuscript and figures:

1) Some figures were merged, and one new figure presenting new data was included. The manuscript now comprises five figures.
2) The quality of all figures has been improved.
3) A new experiment was performed to validate that WT PSTVd can carry certain mutants with nearly correct structure in systemic trafficking. Please refer to Fig 5.
4) Unique sequences and information related to them were presented in a new S2 Table.
5) All raw data have been deposited in NCBI SRA.
6) The deep sequencing data have been further analyzed to examine the composition of mutated non-WT purine/pyrimidine pairs and the occurrence of pseudorevertants in stem regions. Please refer to S2 Fig.
7) The text has been extensively revised to avoid drawing conclusions from PSTVd to other viruses

Review 2 suggests presenting deep sequencing data from 70 libraries on the secondary structure of PSTVd. However, creating 70 figures and a lengthy text to describe and discuss the data would be impractical for a single paper and is beyond the scope of the present study. This study predominantly targets specific stem and loop regions, utilizing sequencing data obtained from saturated mutagenesis within these areas. Consequently, the majority of identified mutations are concentrated within these targeted regions. Alternatively, we have provided all raw data and information on unique sequences from each library. Researchers can access this data for further analyses.

Please check below for our response to each comment:

Reviewer's Responses to Questions

**Part I - Summary**
Please use this section to discuss strengths/weaknesses of study, novelty/significance, general execution and scholarship.

Reviewer #1: In this manuscript, Wu et al. analyzed the changes in 3-D structural diversity of PSTVd mutant populations that occurred during the process in which the mutant pools with locally introduced random mutations spread from the infected cells to surrounding tissues and then to the upper leaves. As the result, the authors found that PSTVd mutant populations is subject to tertiary structure constraints during cell-to-cell movement and long-distance transport, and discussed that their quasispecies composition is shaped by these selective pressures.
The experiment is scientifically designed, and the progress of the experiment and the results obtained are explained in detail in a scientific manner.

The following points can be improved.

Page 9; “a gradual decrease in the number of unique sequences was observed from the pool library to the IR and LM samples”

Comment; In this experiment, at least the number of unique sequences was higher in IR than in pool library, so this statement may be misleading.
Reply-

Lines 256-257, the sentence “a gradual decrease in the number of unique sequences was observed from the pool library to the IR and LM samples” has been modified to read “a decrease in the number of unique sequences was observed from the IR library to the LM samples”
Page 16, L12-L17; “It was observed that certain trafficking-defective mutants (yellow shadow), although they generally accumulated to lower levels than trafficking-competent (red color) mutants, were present in all three replicates of Sys samples across the three loops (S4-S6 Tables), suggesting that trafficking-defective mutants in the PSTVd quasispecies can also be carried by their sister genomes during systemic trafficking”
Comment; Considering that most of the detected trafficking-defective mutants and trafficking-competent mutants differ by only one or two nucleotides, isn’t it possible that these trafficking-defective mutants were derived from trafficking-competent mutants by spontaneous mutations during replication in remote cells?

Reply-

Since we have three biological replicates (Sys 1-3), and the sequencing depth is not very high (only about 50,000 reads for each library), spontaneous mutations are very unlikely to present in all three replicates, especially for the ones with two or more mutations.

Legends for Figures, especially Fig 3, Fig 4, and Fig 5, are very long and seem to overlap with descriptions in the main text. It is preferable to avoid redundant explanations and simply state the necessary matters.

Reply-

We have shortened Figure legends.

Reviewer #2: I approach this review as an RNA virus, but not viroid, specialist.

In this manuscript the authors perform saturation mutagenesis of 7 regions of the potato spindle tuber viroid and then use deep sequencing of input, inoculated region, margins of inoculated leaves, and systemically infected leaves to see which mutants are replication and/or movement competent. The authors also compare how similar the surviving mutants are to WT, at each stage, using Watson-Crick (in stems) and 3D RNA structure (in loops) metrics.

Overall this is a very nice manuscript. The techniques are elegant, I think that the data were analysed in a rigorous and thoughtful manner, and the manuscript is well-written and well-illustrated.

Reviewer #3: In this work Wu and colleagues used mutant pools of the viroid PSTVd to study the role of mutations on the systemic trafficking and infectivity of this sub-viral pathogen. Previous (similar) work from the late Professor Ding’s lab identified several motifs in the PSTVd genomic RNA that were basic for systemic trafficking and replication (10.1105/tpc.107.056606 and 10.3390/v10040160). Here the authors used a similar strategy, but instead they “challenged” a mutant pool and used high-throughput sequencing to understand the final outcome of the host-selected sequences in different types of tissues (which involve cell-to-cell and systemic trafficking). Overall the work is interesting, although it lacks experimental validation of the sequencing data and it could overall analyze the already produced data to a greater extent.

**Part II – Major Issues: Key Experiments Required for Acceptance**
Please use this section to detail the key new experiments or modifications of existing experiments that should be absolutely required to validate study conclusions.

Generally, there should be no more than 3 such required experiments or major modifications for a "Major Revision" recommendation. If more than 3 experiments are necessary to validate the study conclusions, then you are encouraged to recommend "Reject".

Reviewer #1: Not applicable

Reviewer #2: o Basically every one of the 7 sets of mutated regions produces the same phenotype (a gradient of a reduction in the number of surviving sequences from Pool to IR to LM to Sys). The authors' interpretation is that every one of the mutated regions is involved in cell-to-cell and systemic movement. I wonder if this might be an over-interpretation, or perhaps too vague an interpretation to be useful (for example, it may be that every single nucleotide in the genome would show the same phenotype)? Is it possible that some of these movement phenotypes may be due to indirect effects (e.g. reduced stability of the molecule, reduced but not abolished replication, etc) rather than a specific direct role in movement? Related to this above point, there is also a time-course dimension to these samples as IR, LM and Sys were collected at different time points (5, 10 and 12 dpi), thus a reduced rate of replication or increased susceptibility to host defence, for example, might similarly lead to reduced representation in the Sys samples. Thoughts? No new experiments needed, but I suggest toning down the interpretation and/or discussing more what the interpretation really means.
Reply-

We have made the following modifications:

1, line 483-484, the sentence “our comprehensive analysis of the progeny from the L15 mutant pool has revealed that this loop also participates in cell-to-cell movement and long-distance trafficking.” has been rewritten to read “most mutants in this loop were lost after cell-to-cell movement and long-distance”

2, the sentence “This is evident as the mutants which exhibit a structure similar to the WT are preferentially accumulated after these two processes. ” has been deleted.

3 lines 488-490, the sentence “Interestingly, a significantly increased weighted average cutoff score was observed in both the L6 and L27 loops from LM samples to Sys samples, suggesting these two loops also participate in long-distance trafficking of PSTVd.” has been shortened to read “In addition, a significantly increased weighted average cutoff score from LM samples to Sys samples was observed for both L6 and L27.”

4. lines 491-493, the sentence “Our analysis of progeny derived from the mutant pool of L1 showed that this loop is also involved in both cell-to-cell movement and long-distance trafficking.” has been rewritten to read “After cell-to-cell movement and long-distance trafficking, there was a significant decrease in the sequence diversity of PSTVd quasispecies derived from the L1 mutant pool.”

5. lines 493-498, the following sentences have been included: “Thus, our data suggest that all four loops may directly or indirectly affect the cell-to-cell movement and long distance trafficking of PSTVd. Indirect effects may include reduced replication and/or RNA stability. Furthermore, since samples from the IR, LM, and Sys were collected at different time points, a decreased replication rate or increased susceptibility to host defense mechanisms might lead to reduced sequence diversity in Sys samples.”

o The proposal that some movement-defective mutants can be complemented by WT viroid is interesting but, as the authors discuss (p17), the mechanism underlying this is not obvious. It would be useful to at least more directly test this result (e.g. by coinfecting some of these mutants with and without WT viroid to check for complementation) to distinguish between a true complementation effect, as opposed to just the NGS approach being more sensitive to picking up some low level systemic movement of a movement-inhibited mutant.

Reply-

Following your suggestion, we conducted co-infection experiments using both WT and four mutants, encompassing those with positive and negative cutoff scores. Please refer to Fig. 5 and the corresponding text from line 461 to line 481. All four mutants in our study were found to be trafficking-defective. While WT successfully transported a mutant with a positive cutoff score, the other three mutants did not participate in systemic trafficking. Our data suggest that WT can indeed carry certain mutants with a nearly-correct structure in systemic trafficking.

Reviewer #3: Major Issues:

-Availability of raw data in public repositories. High-throughput sequencing data does not seem to be uploaded to a public repository, which is mandatory for publication in any journal. This is also key a service to the scientific community for the potential re-analysis of the author’s data.
Reply-

We have submitted the raw data to NCBI SRA (PRJNA1049893).

Lines 221-223, “The deep sequencing data generated in this study has been deposited in the NCBI Sequence Read Archive (SRA) under the accession number PRJNA1049893.”

-Overall the paper lacks a proper validation of the sequencing data. Neither of the PSTVd mutants identified was tested for its proper systemic trafficking abilities or maintenance of 3D structure.

Reply-

As presented in Tables S5-S7, the systemic trafficking of a large number of mutants identified in the present study has been analyzed by the Ding lab. A considerable number of trafficking-defective mutants were found in the systemic leaves of this study.

As shown in the new Fig. 5, we selected two mutants with a positive cutoff score and two mutants with a negative cutoff score to further test their systemic infection. It was found that all of them exhibited trafficking defects. In mix infection with WT, a mutant with a positive cutoff score was detected in two out of three plants. Our data strongly suggest that WT can carry certain mutants, most probably those with correct or nearly-correct structure, in systemic infection.

As for validating the 3D structure:

1) Loops are closed by Watson-Crick base pairs, and mutations in the loop region generally have no significant impact on the local secondary structure. The present study aligned all mutants on the loop region to the WT structural model and measured their compatibility (or maintenance of 3D structure) using cutoff scores.
2) As we mentioned in the text (lines 226-228), the majority of mutants identified from stem libraries are single or double mutants, typically with minimal effects on the local secondary structure. Also, to minimize the effects of mutations on the local secondary structure, degenerate sites were strategically placed alongside unmutated sites (lines 302-304). The maintenance of 3D structure on stem regions was tested by analyzing the mutated group 1 and group 2 base pairs.

-Another surprising lack of information in the manuscript is a further analysis of the sequencing results. The authors should consider including a map and analysis indicating the location of the identified mutations (including their presence within the already described domains in the PSTVd genome) that are important for the maintenance of both systemic trafficking and the 3D structure of the viroid circular form. This is key to visualize the location of mutations within the very characteristic PSTVd secondary structure.
Reply-

Following the comments from reviewer 3, we conducted a more in-depth analysis of the sequencing data. This included an examination of the composition of non-WT purine/pyrimidine base pairs (S2 Fig) and the identification of pseudorevertants in each library.

This study predominantly targets specific stem and loop regions, utilizing sequencing data obtained from saturated mutagenesis within these areas. Consequently, the majority of identified mutations are concentrated within these targeted regions. Visualizing the location of mutations on the PSTVd secondary structure and conducting additional analyses posed challenges due to the inclusion of 70 libraries in this study. Including 70 separate images in a paper, along with describing and discussing the data presented in those images, may not be practical. However, we have submitted our raw data to NCBI SRA, and the unique sequences identified from each library, along with their corresponding reads number, are provided in S2 Table. Researchers can download this data to perform further analyses.

-Overall quality of the figures. This is a somewhat minor issue, but the constant presence of compression artifacts in the figures and the lack of care in their preparation (boxes overlapping with images/text, PCR screenshots extremely pixelated even in the tiff version of the figures) led to me to include this comment here.

Reply-

All figures have been enhanced. Some RNA blot images may still appear pixelated, a common occurrence, particularly with weak signals.

**Part III – Minor Issues: Editorial and Data Presentation Modifications**
Please use this section for editorial suggestions as well as relatively minor modifications of existing data that would enhance clarity.

Reviewer #1: Not applicable

Reviewer #2: o p2: "could be a more effective approach for antiviral strategies" -> "could be an effective approach for antiviral strategies"

Reply-

Line 53-55, this setence has been rewritten to read “These findings offer insight into viroid quasispecies dynamics and identify RNA 3D structure as a promising avenue for resistance strategies.”

o p9 line 7, text "IR samples, which mimics a replication plus cell-to-cell movement system" - is this the intended text, or are the IR samples supposed to measure replication _without_ a cell-to-cell movement component?

Reply-

This is the intended text: Since PSTVd replicates within cells and moves out of the cells where it replicates to infect new cells, the infected region (IR) samples represent a system encompassing both replication and cell-to-cell movement.
o p10 line 9: Should "mutated base pairs in unique i," read "mutated base pairs in unique sequence i,"?
Reply-

Line 266. This error has been fixed. The sentence now read "mutated base pairs in unique sequence i,".

o In Fig 1a in the inset at left, the "|"s are not aligned properly. Similarly for Fig 4.

Reply-

Figures have been modified to fix these errors.

o It would be useful to some curious readers to add a supplementary table showing the actual sequences of the top 20 unique sequences per region (Fig 3B, Fig 6B) along with their counts-per-1000. This would help interpretation of the results for people interested in the detail.

Reply-

A new S2 Table has been included. This table displays all unique sequences identified from each library. The number of reads, normalized read counts per thousand for each sequence, and cutoff scores for each sequence identified from the loop regions were also presented.

o Consider using a log y-axis scale for Fig 3B and Fig 6B, so that it would be possible to see the read counts for anything other than the WT viroid.

Reply-

According to your suggestion, we re-plotted the data on a log2 scale. Since some figures have been merged, please check Fig. 2B and Fig. 4B.

o In the stem mutants, are G:U pairs overrepresented among the non-WT purine/pyrimidine pairs that survive selection?

Reply-

Following your suggestion, the composition of mutated non-WT purine/pyrimidine pairs was analyzed. Reads of eight mutated purine/pyrimidine pairs (AU, UA, GC, CG, AC, CA, GU, and UG) in all IR, LM, Sys, and pool libraries were counted using the formula RC = Σ(N_i * R_i), where RC represents the read count, N_i represents the number of occurrences of each of the eight purine/pyrimidine pairs in unique sequence i, and R_i represents the total number of reads of unique sequence i. The percentage of RC for each of the eight base pairs in the total RC of all purine/pyrimidine pairs was calculated. Data for IR, LM, and Sys were presented as mean ± SD of three biological replicates, and data for pool libraries were expressed as a single value. However, compared to the pool libraries, no significant changes in the percentage of each of the eight bases were observed in all IR, LM, and Sys samples.

Please refer to S2 Fig and the corresponding text from line 282 to 290.

o Did the authors look for pseudorevertants - especially for the stem mutants, were there any cases where Watson-Crick base-pairings were restored by mutation on the opposite strand from the strand that was originally mutated? This could add extra weight to the 'maintaining WT structure' thesis.

Reply-

Following your suggestion, mutations in all Watson-Crick base pairs (GC, CG, AU, and UA) of S3, S15, and S26 in all libraries were analyzed to identify unique sequences containing a replacement of one base pair by any of the other three. To exclude PCR and sequencing errors, only the top 100 most frequently observed unique sequences from S3 and S15 libraries, and the top 50 most frequently observed unique sequences from S26 libraries (S26 libraries have many fewer unique sequences) were analyzed. However, no such pseudorevertants were observed.

o Regarding the text on p16 "except for one case in L27 (mutant 5'-UUAUAA-3'), all trafficking-defective mutants identified in all three biological replicates of the Sys samples had a positive JAR3D cutoff score": There are very few negative JAR3D scores in Tables S4-S6, so is the all-except-one absence of negative scores statistically significant or just random chance (Fisher's/chi-squared test)?
Reply-

We define those found in all three Sys libraries as carryable mutants, while others are considered non-carryable.

In the case of loop 6 (S5 Table), among the 41 trafficking-defective mutants, 32 have a positive cutoff score, and 9 have a negative score. All mutants with a negative score are non-carryable. Among the 32 mutants with a positive cutoff score, 23 are carryable, and 9 are non-carryable. The Fisher exact test p-value is < 0.01.

For loop 15, among the 18 trafficking-defective mutants, 15 have a positive cutoff score, and 3 have a negative score. All mutants with a negative score are non-carryable. Among the 15 mutants with a positive cutoff score, 13 are carryable, and 2 are non-carryable. The Fisher exact test p-value is < 0.05.

No statistical analysis was performed on loop 27 since all mutants, including the one with a negative cutoff score, are trafficking-competent. However, the one with a negative score has the highest read count in the pool library and a relatively low read count in Sys libraries.

Lines, 447-460, the following description has been included- “Mutants found in all three Sys libraries were defined as carryable mutants, while others are considered non-carryable. In the case of loop 6 (S5 Table), among the 41 trafficking-defective mutants, 32 have a positive cutoff score, and nine have a negative score. All mutants with a negative score are non-carryable. Among the 32 mutants with a positive cutoff score, 23 are carryable and nine are non-carryable. For loop 15 (S6 Table), among the 18 mutants, 15 have a positive cutoff score, and three have a negative score. All mutants with a negative score are non-carryable. Among the 15 mutants with a positive cutoff score, 13 are carryable, and two are non-carryable. Fisher’s exact test confirmed that in both the S6 and S15 libraries, trafficking-defective mutants with a positive cutoff score are more likely to be carried and reach Sys tissues (p < 0.01 and 0.05, respectively). No statistical analysis was performed on loop 27 since all mutants, including one with a negative cutoff score, are trafficking-competent (S7 Table). However, this mutant had the highest read count in the pool library and a relatively low read count in Sys libraries.”

o On p12, I don't really understand what is meant by the text "our findings suggest that RNA 3D structure can _drive_the_sequence_organization_ of PSTVd stem regions primarily through cell-to-cell and long-distance trafficking" and "Together, these selective processes can lead to the _reorganization_ of sequences within PSTVd quasispecies." Consider rewording.

Reply

Lines 335-336, the sentence “Taken together, our findings suggest that RNA 3D structure can drive the sequence organization of PSTVd stem regions primarily through cell-to-cell and long-distance trafficking. In addition, the effect of long-distance trafficking on sequence diversity is more important than cell-to-cell movement. ” has been modified to read “Taken together, our findings suggest that PSTVd variants in stem regions with correct or nearly correct RNA 3D structure are more capable of cell-to-cell and long-distance trafficking.”.

Lines 343-344, the sentence “Together, these selective processes can lead to the reorganization of sequences within PSTVd quasispecies.” has been modified to read “Together, these selective processes can shape the sequence organization of PSTVd quasispecies.”

o On p12, the text on RNA virus movement is not really relevant to viroid movement since RNA plant viruses all encode movement proteins. Consider deleting or moving to Discussion?

Reply-

We deleted the following sentences: “The mechanisms responsible for the intercellular movement and long-distance systemic trafficking of RNA viruses differ significantly. Movement of RNA viruses between cells through plasmodesmata is a rapid process that occurs within a few hours.”

Lines 337-338, the sentence “On the other hand, phloem loading, which marks the initial step of long-distance trafficking, can take several days due to the complex structure of plasmodesmata pore units and the involvement of various factors, including multiple viral proteins” has been modified to read “Studies on both viroids and viruses suggest that plasmodesmal gates between different cell types are unique and unidirectional [26-32, 51].”

o Some of the generalisations to RNA viruses (as opposed to viroids) are not supported and should probably be toned down e.g.
- p17 "Our study also provided novel insights into the constraints on sequence diversity within the quasispecies of viruses"
- p3 "Gaining insights into viroid quasispecies evolution is highly significant as it improves our understanding of viral evolution and quasispecies structure."
- p2 "These findings emphasize the potential of targeting RNA 3D structure as a more robust approach for antiviral strategies."

Reply-

The sentence “Our study also provided novel insights into the constraints on sequence diversity within the quasispecies of viruses, as emerging evidence has shown the involvement of RNA structure in viral infection [35-37]” has been deleted.

Lines 79-82, the sentence “Gaining insights into viroid quasispecies evolution is highly significant as it improves our understanding of viral evolution and quasispecies structure. Additionally, it can aid in the development of novel antiviral approaches and advance our knowledge of RNA movement in the host organism.” has been modified to read “Gaining insights into viroid quasispecies evolution is highly significant as it can aid in the development of novel approaches to combat viroid infections and advance our knowledge of RNA movement in the host organism.”

Lines 37-38. the sentence “These findings emphasize the potential of targeting RNA 3D structure as a more robust approach for antiviral strategies.” has been modified to read “These findings emphasize the potential of targeting RNA 3D structure as a more robust approach to defend against viroid infections.”

We have also check the whole manuscript to avoid the generalisations to RNA viruses.

Reviewer #3: Minor issues:

-Lack of information in material and methods about the analysis of the sequencing data. The authors mentioned that “a Java-based pipeline” or a “A Java program” were used to analyze the data. The program in question is not described at all. This is an insufficient amount of information to replicate the authors results and should be explained in much further detail.

Reply-

Lines, 555- 588, this section has been significantly enriched to explain the Java program in details.

-The manuscript contains an unnecessary large number of figures. In my opinion Figures 1 and 2, 3 and 4, and 6 and 7 could be joined. That will leave the manuscript with a very appropriate number of 4 figures.

Reply-

Following your suggestion, we have merged some figures, and with the addition of one more figure displaying new results, the manuscript now comprises a total of five figures.

-In the analysis carried out in Figure 7 the cutoff score of the wt PSTVd sequence should be shown as a reference and to allow a proper comparison of the values.
Reply-

Following your suggestion, cutoff score of WT PSTVd has been included. Please check Fig.4D.

-How could trafficking-defective mutants in the systemic tissues be carried out by functional sister genomes? Has any type of aggregation between viroid genomes been described in the literature? If not supported by the literature, I would guess that those sequences are able to move systemically due to similar interactions with transport proteins or a simple dilution of the mutant from the original infiltration/infection point.

Reply-

It has been well-documented that certain nonfunctional mutants of viruses can be carried both in replication and trafficking by functional proteins produced by their sister genomes. However, whether this phenomenon applies to viroids is unknown. As presented in Tables S5-S7, the systemic trafficking of a large number of mutants identified in the present study has been analyzed by the Ding lab. A conisderable number of trafficking-defective mutants were found in the systemic leaves of this study.

As shown in the new Fig. 5, we selected two mutants with a positive cutoff score and two mutants with a negative cutoff score to further test their systemic infection. It was found that all of them exhibited trafficking defects. This conclusion is based on data from RNA blot and RT-PCR. RT-PCR is a very sensitive method. This implies that these mutants, at the very least, cannot interact similarly with transport proteins or simply dilute from the original infiltration/infection point to systemic leaves by themselves.

In mix infection with WT, a mutant with a positive cutoff score was detected in two out of three plants. Our data strongly suggest that WT can carry certain mutants, most probably those with correct or nearly-correct structure, in systemic infection.
